# Adverse childhood experiences, resilience and mental health among young people aged 16–29 years in Nepal: a population-based household survey (2025)

**DOI:** 10.1016/j.lansea.2026.100803

**Published:** 2026-06-15

**Authors:** Subash Thapa, Nancy Ross, Santosh Giri, Kedir Y. Ahmed, Shivani Bhattarai, David G. Fogden, Alisha Basnet, Julaine Allan

**Affiliations:** a Rural Health Research Institute (RHRI), Charles Sturt University, Orange, NSW, 2800, Australia; b School of Social Work, Dalhousie University, K'jipuktuk Halifax, Nova Scotia, Canada; c Discipline of General Practice, School of Medicine, Adelaide University, Adelaide, SA, Australia; d Adventist Development and Relief Agency (ADRA), Lalitpur, 44700, Nepal; e Danish Red Cross-Nepal and Bangladesh, Kathmandu, 44600, Nepal; f Health Inequality and Resilience Research Institute, Kathmandu, 44600, Nepal

**Keywords:** Adverse childhood experiences, Mental health, Nepal, Psychological distress, Resilience, Social support, Suicide attempts, Young people

## Abstract

**Background:**

Mental health conditions are a major public health challenge for young people globally. Although adverse childhood experiences (ACEs) are established risk factors for poor mental health, population burden of ACEs and their association with modifiable psychosocial factors and mental health outcomes among young people remain limited in South Asian countries, including Nepal, thereby constraining the development of evidence-informed prevention strategies. To address this gap, this study examines the associations of ACEs and protective psychosocial factors with mental health outcomes, including depression, anxiety, psychological distress, and suicide attempts among young people in Nepal.

**Methods:**

A cross-sectional survey of Nepali residents aged 16–29 years was conducted using a multi-stage cluster sampling design across three randomly selected provinces, with survey estimates applied to generate population-level estimates. An interviewer administered questionnaire was used to collect data on socio-demographic factors, ACEs, psychosocial characteristics, health behaviours, and mental health outcomes. ACEs were examined as a cumulatively and by domain, along with resilience and perceived social support assessed as potential protective factors. Weighted multilevel logistic regression models were used to estimate the odds ratios (ORs) for associations with mental health outcomes.

**Findings:**

The study included a weighted sample of 6055 individuals, with mean age of 21.1 (±4.1) years and 54.3% were female. A total of 62.4% reported exposure to at least one ACE, with 10.4% experiencing four or more. The overall prevalence of anxiety was 24.3% (95% CI: 23.2–25.4), depression was 6.0% (95% CI: 5.4–6.6), psychological distress was 9.8% (95% CI: 9.0–10.6) and suicide attempt was 1.9% (95% CI: 1.6–2.3), with the rates higher among female participants. Those with four or more ACEs had significantly higher prevalence of anxiety (44.9%), depression (15.4%), psychological distress (26.4%), and suicide attempts (9.48%). Individuals with four or more ACEs were associated with increased odds of anxiety (OR = 2.72; 95% CI: 2.05–3.62), depression (OR = 2.61; 95% CI: 1.74–3.91), psychological distress (OR = 4.67; 95% CI: 3.22–6.77), and suicide attempts (OR = 9.60; 95% CI: 4.44–20.75). Those with lower household wealth, current smokers and belonging to the female sex were associated with higher odds of anxiety, depression, and psychological distress. Perceived social support accounted for 14.2%, 26.1%, 13.8%, and 21.6% of the associations between ACEs and anxiety, depression, psychological distress, and suicide attempts.

**Interpretation:**

The findings suggest that addressing childhood adversity is important and may contribute to reduction of the burden of mental health conditions among young people in Nepal. ACEs screening and prevention, alongside interventions that strengthen resilience and promote social support, are urgently needed, particularly for young women and economically disadvantaged young populations.

**Funding:**

This work was funded by a grant from the 10.13039/501100001769Charles Sturt University, Australia.

**Research in context**

**Evidence before this study**

Mental health conditions represent the leading cause of disability among young people globally. Adverse childhood experiences (ACEs), including maltreatment, neglect, and family dysfunction, are established as major contributors to the global burden of disease and disability. International evidence demonstrates that exposure to ACEs at any developmental stage is associated with marked increases in risk for mental health conditions, including depression, anxiety, distress and suicidal behaviour. While a growing body of literature documents associations between ACEs and mental health problems in low- and middle-income countries, population-level data specifically examining the burden of ACEs among young people in Nepal and other South Asian nations are limited. Furthermore, resilience factors and protective mechanisms, such as social support, have been identified as critical mediators of the effect of ACEs on mental health outcomes in other populations, but their role remains underexplored in Nepali youth.

**Added value of this study**

This population-based study quantifies the burden of ACEs and their associations with depression, anxiety, psychological distress, and suicide attempts among young people aged 16–29 years in Nepal. By examining a weighted sample of 6055 individuals, this study provides population-level evidence on modifiable socioeconomic and behavioural risk factors (lower household wealth, smoking) and psychosocial factors (resilience and perceived social support) that could inform targeted prevention and intervention strategies. The dose–response relationship observed between ACE exposure and mental health outcomes, particularly the substantial increase in odds ratios with four or more ACEs (anxiety OR = 2.72, depression OR = 2.61, psychological distress OR = 4.67, suicide attempts OR = 9.60) provides compelling evidence for the clinical significance of childhood adversity in this population. Additionally, the study identifies vulnerable subgroups (young women and those in low-income households) who may benefit from enhanced prevention efforts. Such interventions should emphasise enhancing individual resilience and perceived social support, as these factors were shown to act as mechanisms linking ACEs to anxiety, depression, psychological distress, and suicide attempts.

**Implications of all the available evidence**

Our findings suggest that even modest reductions in ACE exposure could yield substantial population-level improvements in mental health outcomes. To reduce the burden of depression, anxiety, distress and suicidal behaviour among Nepali adolescents and young adults, integrated interventions must target both ACE prevention and the promotion of protective factors. Current mental health policies in Nepal should prioritise: (1) early identification and screening for childhood trauma within primary care and school-based settings; (2) resilience-building and psychosocial support interventions, particularly for economically disadvantaged youth; (3) addressing upstream socioeconomic determinants, including poverty and limited access to health services; and (4) strengthening community-level protective mechanisms through enhanced social support networks and culturally-informed mental health services. The government should increase investment in evidence-based ACE prevention and mental health promotion programmes that engage young people, families, and communities in Nepal's ongoing health system strengthening efforts.

## Introduction

Mental health conditions among young people represent a growing global public health challenge, with an estimated 279 million adolescents and young adults living with mental health conditions in 2021, contributing substantially to disability, social disruption, and economic burden worldwide.^1^^,^^2^ Adolescence and early adulthood (16–29 years) are important developmental periods, during which exposure to life stressors may increase vulnerability to mental health conditions, such as anxiety, depression and suicidal behaviour.^3^ These disorders, if untreated, may lead to long-term morbidity and wide-ranging functional impairment throughout the life course.

Emerging evidence indicates that prolonged exposure to early life trauma and stress can induce lasting biological, cognitive, and psychological changes, as well as disrupt attachment and social development, increasing susceptibility to mental health conditions in adolescence and adulthood.^4^^,^^5^ Traumatic events occurring before age 18, known as “Adverse Childhood Experiences (ACEs),” include physical, emotional, and sexual abuse; neglect; household dysfunction such as parental mental illness, incarceration, or substance use; parental divorce; and environmental stressors such as bullying, community violence, and natural disasters.^4^^,^^5^ Although evidence from higher-income countries (HIC) has primarily established the link between ACEs and poor mental health, population-level assessments of ACEs and their impact on mental health conditions research in low- and middle-income countries (LMICs), particularly across South Asia, remains sparse.^6^

As a result, a public health approach to address ACEs has generally not been integrated into national health policies in Asia, except in a few countries such as Thailand, where ACE-informed mental health prevention exist.^7^^,^^8^ The lack of population-based data represents a major evidence gap, hindering the development of trauma-informed interventions and preventive strategies.

Nepal is experiencing an increasing burden of mental health conditions, particularly among young people, with one in ten young people aged 18–29 living with a mental condition.^9^, ^10^, ^11^ Previous studies in selective populations, such as children rescued from child labour, hospital patients, or children from socioeconomically disadvantaged communities, have shown that ACEs increase the risk of mental health conditions, especially among female participants, rural populations, and low-income groups.^12^, ^13^, ^14^, ^15^, ^16^, ^17^, ^18^, ^19^, ^20^ However, most studies in Nepal remain small-scale and region-specific, and have not comprehensively assessed the full spectrum of ACE domains, including household dysfunction, neglect, or community-level adversity, such as bullying.

Additionally, little is known about whether potentially modifiable psychosocial factors can attenuate the impact of ACEs on mental health among young people in a resource limited settings In our recent Australian study, we demonstrated that higher levels of perceived social support and personal mastery substantially buffer the adverse effects of early life adversity on psychological distress, depression, and anxiety.^21^ These findings highlight the role of key resilience-enhancing mechanisms to enable individuals to adapt to and recover from stress and underlying mental health conditions. Integrating both risk and protective factors within a unified analytical framework is essential for identifying potentially modifiable psychosocial mechanisms and for informing culturally grounded, trauma-informed prevention and intervention strategies.^21^

Although Nepal's National Mental Health Strategy (2020) recognises adolescent mental health, none of the country's mental health policy documents clearly address childhood trauma or ACEs prevention and management.^11^ There is also a critical need to scale up mental health services, integrate trauma-informed care into routine care, and implement targeted ACE prevention programs for youth exposed to childhood adversities. To address this gap, this study examines the associations of ACEs and protective psychosocial factors with mental health outcomes, including depression, anxiety, psychological distress, and suicide attempts among young people in Nepal. We also investigate whether the effects of ACEs on mental health outcomes are mediated by perceived social support and resilience.

## Methods

A population-based cross-sectional survey of young people aged 16–29 years was conducted in Nepal between March and September 2025 across three randomly selected provinces of Nepal. The selected provinces (Bagmati, Madhesh, and Sudurpaschim) capture substantial geographic and socioeconomic diversity within Nepal. Bagmati includes the capital region and predominantly urban populations; Madhesh represents lowland Terai regions with higher population density and cross-border mobility; and Sudurpaschim includes more remote and economically disadvantaged areas. Together, these provinces encompass urban and rural settings, diverse ethnic compositions, and varying socioeconomic conditions.

**Fig. 1:**
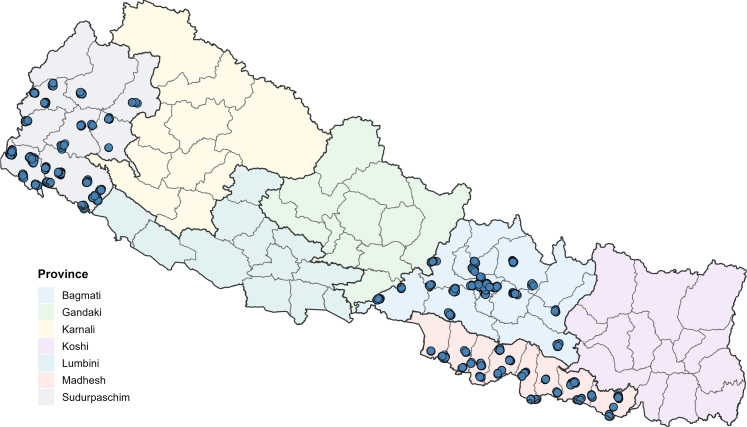
Study sites.

Nepal is a lower-middle-income country in South Asia with a population of approximately 30 million. Administratively, Nepal comprises 7 provinces, 77 districts, and 753 local government units (metropolitan cities, sub-metropolitan cities, municipalities, and rural municipalities). This administrative structure informed the multi-stage probability sampling framework. Wards, the smallest administrative and service-delivery units, served as primary sampling units (Fig. 1).

The source population comprised all young people aged 16–29 years residing in Nepal. The study population consisted of individuals from this source population who were living in the randomly selected wards at the time of data collection. Participants were eligible if they were Nepali residents aged 16–29 years and had the cognitive ability to provide informed consent and participate in a face-to-face interview, as assessed subjectively by trained enumerators.

**Fig. 2:**
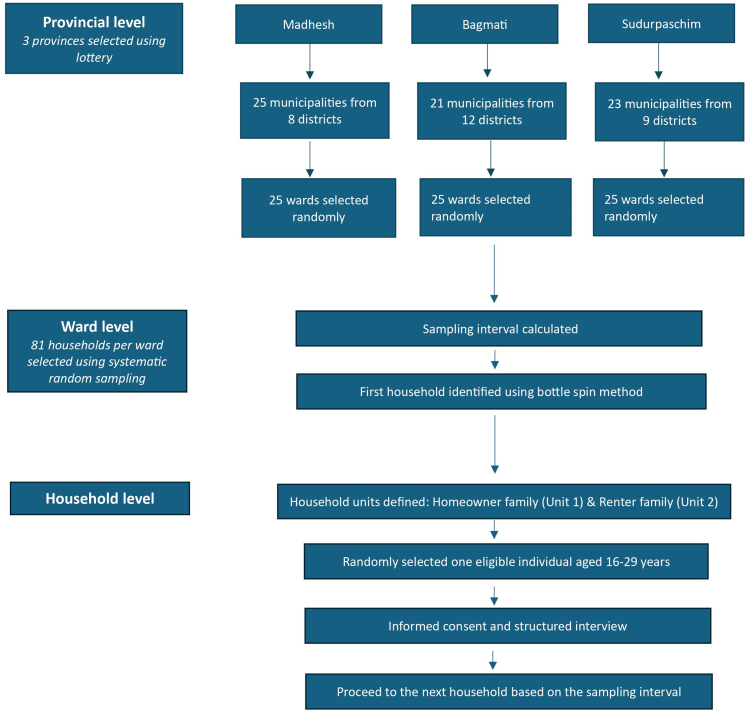
Sampling flow diagram.

A multi-stage cluster sampling design was used to select the study participants (Figs. 1 and 2). In the first stage, three of Nepal's provinces (Bagmati, Madhesh, and Sudurpaschim) were selected through simple random sampling. In the second stage, wards were selected directly from the complete list of wards within each selected province using probability proportional to size (PPS), based on the population and household estimates from 2021 national census. Municipal boundaries were not treated as an intermediate sampling stage. In the final stage, households within selected wards were selected using systematic random sampling.

Within each selected ward, a household listing was first constructed to identify all households containing at least one eligible individual aged 16–29 years. This list constituted the sampling frame for the final stage of selection. The sampling interval (K) was calculated by dividing the total number of eligible households in the ward by the target sample size for that ward. A random starting point between 1 and K was selected, and every K^th^ household was approached thereafter until the required number of households was reached. In circumstances where household was locked, vacant, or refused participation, the next household on the list was approached to maintain the sampling interval.

In households comprising multiple families functioning as separate economic units, each unit was considered independently, and one was randomly selected. In households with multiple eligible participants aged 16–29 years, one participant was randomly selected using a lottery method. If the selected participant was unavailable at the time of visit, up to three follow-up visits were scheduled. Household visits were conducted on all days of the week between 06:30 and 20:00 h.

Sampling weights were calculated as the inverse of the probability of selection at each sampling stage (province, ward, household, and individual) and adjusted for household nonresponse within each ward. The overall inclusion probability was obtained by multiplying stage-specific probabilities. All analyses incorporated these weights to generate population-level estimates and to account for the multi-stage cluster design. Survey analyses accounted for stratification by province and area of residence, with clustering specified at the ward (primary sampling unit) level using Taylor-linearised variance estimation with single-unit strata centred. Weighted and unweighted estimates were comparable, suggesting minimal distortion from unequal selection probabilities.

Of 6100 households visited, 6055 individual interviews were completed (response rate: 99.3%). Forty-five households (0.7%) declined participation, and 49 households (0.8%) required a second visit to complete the interview.

Face-to-face interviews were conducted using a structured questionnaire in Nepali, administered via Open Data Kit (ODK) on computer tablets. The questionnaire covered socio-demographic information, health behaviours, adverse childhood experiences (ACEs), resilience measures, and mental health history (see Table 1). Physical measurements, including height and weight, were collected to calculate body mass index. The questionnaire was developed in English and translated into Nepali. A research team including consultant psychiatrists and mental health researchers from Nepal reviewed the questionnaire to ensure content validity, clarity, and relevance to study objectives. Pre-testing was conducted with 100 individuals aged 16–29 years in rural communities of Lalitpur district (outside the study areas) in November 2024 to assess wording, response categories, and question order.

**Table 1:** Study variables.

Variables	Description
**Outcomes**	
Depression	The Patient Health Questionnaire-9 (PHQ-9) is a widely used screening tool for depressive symptoms. It consists of 9 items rated on a 4-point Likert scale (0 = Not at all to 3 = Always).^22^^,^^23^ Scores range from 0 to 27. A cutoff score of 10 or more indicates clinically significant depression using thresholds used in National Demographic and Health Survey 2022.^24^ Categorised as: 0 = No depression, 1 = Depressed
Anxiety	The Generalised Anxiety Disorder scale (GAD-7) assesses anxiety symptoms over the past two weeks. It includes 7 items rated on a 4-point Likert scale (0 = Not at all to 3 = Always).^23^ Scores range from 0 to 21. Consistent with thresholds used in National Demographic and Health Survey 2022, a cutoff score of 6 or more indicates the presence of anxiety symptoms rather than clinically diagnosed anxiety disorder.^24^ Categorised as: 0 = No anxiety, 1 = Anxious
Psychological distress	The Kessler Psychological Distress Scale (K-6) measures nonspecific psychological distress. It includes 6 items rated on a 5-point Likert scale (0 = none of the time to 4 = all of the time).^25^ Scores range from 6 to 30. A cutoff score of 16 or more indicates high distress. Categorised as: 0 = No distress, 1 = Distressed
Suicide attempts	Based on the question: “During the past 12 months, how many times did you attempt suicide?” Responses were recoded into binary categories: 0 = No attempt, 1 = One or more attempts
**Explanatory variables**	
ACEs	ACEs were measured using a set of items adapted from the WHO ACEs-International Questionnaire.^26^ and widely applied in population-based surveys. The instrument included domains covering abuse, neglect, household dysfunction and peer victimisation occurring before age 18, typically measured through a cumulative score of yes/no items. Higher scores indicate greater adversity. Categorised as: 0 = No ACEs, 1 = One ACE, 2 = Two ACEs, 3 = Three ACEs, and 4 = 4+ ACEs. Similar ACE measures have been used in previous studies in low- and middle-income country settings. In addition, ACEs were grouped into four domains (neglect, abuse, household dysfunction, and peer victimisation) to examine which combinations of adversities were most strongly associated with mental health outcomes.
Perceived Social Support	The Perceived Social Support (PSS) scale measures the extent to which individuals feel supported by family, friends, and significant others. It includes 5 items rated on a 5-point Likert scale (1 = strongly disagree to 5 = strongly agree). Total scores range from 6 to 30, with higher scores reflecting stronger perceived support.
Resilience	The Brief Resilience Scale (BRS) assesses the ability to recover from stress.^27^ It includes 6 items rated on a 5-point Likert scale (1 = strongly disagree to 5 = strongly agree). Items 2, 4 and 6 were reverse-coded. The average score across items is used, ranging from 1 to 5. Higher scores indicate greater resilience.
Current smoking	Current smoking behaviour: 0 = No, 1 = Yes
Alcohol	Alcohol consumption in the past month: 0 = No, 1 = Yes
BMI	Body Mass Index calculated from measured height and weight (kg/m^2^). Used as a continuous variable to assess underweight, normal weight, overweight, and obesity.
Place of residence	Type of living area where the participant resides (Rural, Urban)
Education	Highest level of formal education attained (Illiterate/informal, Grade 1–10, High school, Bachelor or higher)
Household income	Household wealth index derived using principal component analysis (PCA) of household assets. Quintiles represent relative economic status (Poorest, Poorer, Middle, Richer, Richest)
**Covariates**	
Age	Age (in years) was included as a continuous covariate in all regression models to account for developmental differences across the study population.
Sex	Biological sex of the participant: 0 = Female, 1 = Male
Marital status	Current marital status of the participant: Single, Married, Divorced/Separated, Widowed

ACEs, Adverse Childhood Experiences.

Enumerators were selected based on public health background (bachelor's degree minimum) and received two weeks of training on handling sensitive topics, ensuring ethical conduct, and using ODK tools effectively. Community-based pretesting helped identify and address issues with the questionnaire and ODK system. Community engagement activities in the selected areas were conducted between December 2024 and February 2025. These activities included consultations with local government officials, health workers and community leaders, as well as sensitisation meetings with female community health volunteers and youth groups to build trust, address stigma related to mental health and facilitate community participation. Household interviews took place between March and September 2025, with each interview lasting approximately 35 min. Interviews were conducted in participants' households in private, safe settings to ensure confidentiality, minimise reporting bias and encourage disclosure of sensitive experiences. Data were submitted daily via tablets to a designated statistician for monitoring and quality assurance.

Data were uploaded daily to a secure server, where real-time monitoring checks identified inconsistencies or missing items. Potential errors were flagged and communicated to the data collection team for immediate correction. Automated routine data quality reports (created using R version 4.4.2) were shared regularly with field supervisors and the monitoring team to ensure protocol adherence. Upon completion of data collection, all records were reviewed and verified directly with data collectors to resolve any outstanding discrepancies.

### Measures

Based on the existing literature, certain forms of adversity, such as harsh parenting, domestic violence, household mental illness, and economic hardship, are more common in Nepalese communities, which informed the selection of variables and the inclusion of specific ACE domains in this study.^15^^,^^28^ Study variables were categorised into outcomes, primary exposure, additional explanatory variables, and covariates (Table 1).

Outcomes included depression, anxiety, psychological distress, and suicide attempts, which are measured using related but distinct constructs (e.g., GAD-7, PHQ-9, and K-6), representing different symptom domains and levels of severity. Analysing these outcomes separately allows examination of whether ACE exposure shows consistent associations across multiple dimensions of mental health, rather than relying on a single composite indicator. The internal consistency of the instruments in the present sample was assessed using Cronbach's alpha coefficients (PHQ-9 = 0.83, GAD-7 = 0.82, K-6 = 0.84, BRS = 0.86, and PSS = 0.76).

The primary exposure of interest was ACEs, measured as cumulative score. Additional explanatory variables included resilience, perceived social support, health risk behaviours (smoking and alcohol use), BMI, place of residence and socio-economic factors such as household wealth index and education. Covariates included age, sex, and marital status.

### Data analysis

At first, raw survey data were checked for completeness, consistency, and outliers. Initial screening included checks for duplicate records, invalid codes, and structural errors. Continuous variables were examined for out-of-range and improbable values using histograms, box plots, and descriptive statistics. Categorical variables were reviewed for misclassified or sparse categories, which were harmonised when conceptually appropriate. Missing data patterns were explored using cross-tabulations and diagnostics to identify systematic missingness. All derived variables, including cumulative ACE scores, socioeconomic indicators, and age-standardised measures, were created using predefined coding rules, with reproducible syntax archived for transparency.

Survey design features were applied throughout using appropriate survey weights to obtain population-representative estimates. Weighted multivariable logistic regression models were fitted to estimate associations between cumulative ACE exposure and each outcome, after adjusting for age, sex, marital status, education, wealth quintile, place of residence, smoking, alcohol use, and BMI. To formally assess the pattern of association, the ACE score was additionally modelled as a continuous variable to test for linear trend, and non-linearity was evaluated by introducing a quadratic term (ACE^2^); a significant quadratic coefficient was interpreted as evidence of an accelerating association. ACE-type specificity was examined by clustering individual ACEs into four domains: abuse, neglect, household dysfunction, and peer victimisation. Effect modification by sex and wealth quintile and age-group heterogeneity (16–19 years vs 20–29 years) were assessed through interaction testing and stratified analyses. Detailed results are presented in Supplementary Tables S1 and S2. Statistical significance was defined as p < 0.05, and all analyses were performed in Stata version 18.0 (StataCorp, USA).

We hypothesised that the association between ACEs and mental health outcomes would be partially mediated by social support and resilience. Mediation analyses were conducted within the counterfactual framework using natural effect models,^29^^,^^30^ implemented in R (version 4.4.0)^31^ using the “medflex package”.^32^ An imputation-based approach was used to decompose the Total Association (TA) into the Direct (DA) and Indirect Association (IA) operating through each mediator.^33^ ACE exposure was dichotomised as none versus any (≥1 ACE) to assess whether the presence of childhood adversity, compared with its absence, operates through social support and resilience to affect mental health outcomes. Given the cross-sectional design, temporal ordering cannot be empirically verified; these analyses are therefore interpreted as exploratory statistical decompositions rather than causal pathways. Results are presented as ORs with 95% CIs, with proportion mediated calculated on the log–odds scale.

### Ethics

Ethical approval was obtained from the Charles Sturt University Human Research Ethics Committee (CSU-HREC reference H24281) and the Nepal Health Research Council Institutional Ethics Review Board (NHRC-IERB reference 450–2024). The study adhered to the Declaration of Helsinki. Written informed consent was obtained from adult participants, while written assent and parental or guardian consent were obtained for participants under 18 years.

### Role of the funding source

Funders had no role in study design, data collection, data analysis, interpretation or writing of the report.

## Results

The final analysis included a total of 6055 participants with mean age of 21.1 (±4.1) years (Table 2). Altogether, 3286 (54.3%) were female and 2769 (45.7%) were male. Most participants were unmarried (69.8%, n = 4220) and completed Grade 1–10 (50.9%, n = 3088). Most participants resided in urban areas (68.2%, n = 4119). The majority reported not currently smoking (82.3%, n = 4979) and not consuming alcohol in the last month (83.8%, n = 5067). A total of 37.6% (n = 2269) had no ACEs, and 62.4% (n = 3786) had at least one ACEs.

**Table 2:** Characteristics of sample population.

	Female (N = 3286)	Male (N = 2769)	Total (N = 6055)
	n (%)	n (%)	n (%)
**Age** (mean/SD)	21.3 (±4.1)	20.9 (±4.0)	21.1 (±4.1)
**Marital status**			
Unmarried	1976 (46.8)	2244 (53.2)	4220 (69.8)
Currently married	1296 (71.8)	511 (28.2)	1807 (29.7)
Divorced/Separated/Widowed	14 (50.1)	14 (49.9)	28 (0.5)
**Education**			
Illiterate/informal education	149 (81.9)	33 (18.1)	182 (3.0)
Grade 1–10	1690 (54.7)	1398 (45.3)	3088 (50.9)
High school	1076 (51.9)	1001 (48.1)	2077 (34.4)
Bachelor's or higher	371 (52.3)	337 (47.7)	708 (11.7)
**Household wealth status**			
Poorest	748 (61.0)	481 (39.0)	1229 (20.0)
Poorer	693 (57.3)	517 (42.7)	1210 (20.0)
Middle	651 (53.5)	566 (46.5)	1217 (20.2)
Richer	631 (53.2)	555 (46.8)	1186 (19.6)
Richest	563 (46.4)	650 (53.6)	1213 (20.1)
**Place of residence**			
Rural	982 (50.7)	954 (49.3)	1936 (31.8)
Urban	2304 (56.0)	1815 (44.0)	4119 (68.2)
**Smoking**			
No	3214 (64.5)	1765 (35.5)	4979 (82.3)
Yes	72 (6.7)	1004 (93.3)	1076 (17.7)
**Alcohol consumption in the last month**			
No	3160 (62.3)	1907 (37.7)	5067 (83.8)
Yes	126 (12.8)	862 (87.2)	988 (16.2)
**Body Mass Index (BMI)**	21.0 (±3.1)	20.9 (±3.1)	20.9 (±3.1)
**Multiple adverse experiences**			
No ACEs	1241 (54.7)	1028 (45.3)	2269 (37.6)
One ACE	867 (52.5)	786 (47.5)	1653 (27.3)
Two ACEs	496 (51.3)	474 (48.7)	970 (16.0)
Three ACEs	306 (57.5)	225 (42.5)	531 (8.8)
4+ ACEs	376 (59.4)	256 (40.6)	632 (10.4)
**Perceived Social Support score** (mean/SD)	23.5 (±4.0)	24.2 (±3.5)	23.8 (±3.8)
**Brief Resilience Scale score** (mean/SD)	3.0 (±0.8)	3.3 (±0.8)	3.1 (±0.8)

Data are n (%) for categorical variables and Mean(±SD) for continuous outcomes.

Among 6055 participants, 62.4% (n = 3781) reported exposure to at least one ACE, whereas 37.6% (n = 2269) reported no ACE exposure (Table 3). The most reported ACEs were household substance use (36.4%), household violence (17.5%), physical neglect (17.1%), physical abuse (14.2%), and verbal abuse (14.2%), while sexual abuse (2.2%) and parental divorce (1.8%) were less common.

**Table 3:** Prevalence of specific adverse childhood experiences (ACEs).

	Total (N = 6100)	Total % (95% CI)	Female % (95% CI)	Male % (95% CI)
Physical abuse	851	14.2 (13.3, 15.1)	11.1 (10.0, 12.2)	17.8 (16.5, 19.3)
Sexual abuse	133	2.2 (1.9, 2.6)	3.7 (3.1, 4.4)	0.4 (0.2, 0.7)
Verbal abuse	856	14.2 (13.3, 15.1)	15.3 (14.1, 16.6)	12.9 (11.7, 14.2)
Physical neglect	1036	17.1 (16.2, 18.1)	18.4 (17.1, 19.8)	15.6 (14.3, 17.0)
Emotional neglect	544	9.0 (8.3, 9.7)	10.6 (9.6, 11.7)	7.0 (6.1, 8.0)
Household violence	1053	17.5 (16.6, 18.5)	18.6 (17.3, 20.0)	16.2 (14.9, 17.6)
Parental divorce	114	1.8 (1.5, 2.2)	2.2 (1.8, 2.8)	1.3 (0.9, 1.8)
Death of a parent	465	7.6 (6.9, 8.3)	8.4 (7.5, 9.4)	6.6 (5.7, 7.5)
Parental mental illness	426	7.0 (6.3, 7.6)	8.3 (7.4, 9.3)	5.3 (4.5, 6.2)
Household substance use	2224	36.4 (35.2, 37.6)	35.9 (34.3, 37.5)	36.9 (35.1, 38.7)
Incarceration	177	2.9 (2.5, 3.3)	3.0 (2.4, 3.6)	2.8 (2.3, 3.5)
Peer victimisation (Bullying)	400	6.6 (6.0, 7.3)	5.1 (4.4, 6.0)	8.3 (7.4, 9.4)

**Fig. 3:**
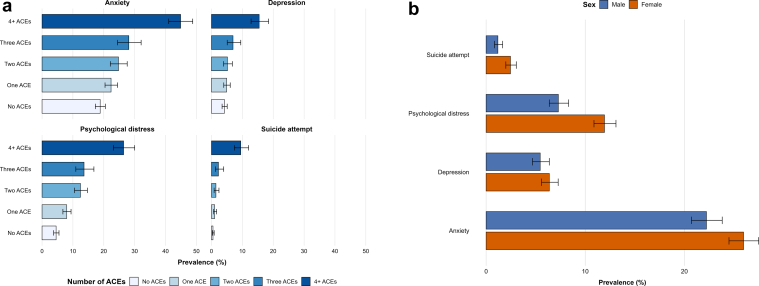
a & b. Prevalence of mental-health outcomes by sex and number of ACEs.

The overall prevalence of anxiety was 24.3% (95% CI: 23.2–25.4), depression was 6.0% (95% CI: 5.4–6.6), psychological distress was 9.8% (95% CI: 9.0–10.6) and suicide attempt was 1.9% (95% CI: 1.6–2.3) (Fig. 3a). The prevalence of anxiety increased from 18.8% among those with no ACEs to 44.9% among those with four or more ACEs (Fig. 3b).

Multivariable logistic regression models adjusted for demographic, socioeconomic, and behavioural covariates are presented in Table 4. Men were less likely to experience anxiety compared with women (OR = 0.68; 95% CI: 0.55–0.83). Participants who were divorced, separated, or widowed were more likely to experience anxiety compared with unmarried participants (OR = 3.34; 95% CI: 1.51–7.37). Lower household wealth was associated with higher anxiety. Current smokers were more likely to experience anxiety (OR = 2.37; 95% CI: 1.73–3.25) than non-smokers. Those with exposure to four or more ACEs were more likely to experience anxiety (OR = 2.72; 95% CI: 2.05–3.62) than those without ACEs. Those with higher perceived social support (OR = 0.97; 95% CI: 0.95–0.99) and greater resilience (OR = 0.79; 95% CI: 0.70–0.91) were associated with lower likelihood of experiencing anxiety (Table 4).

**Table 4:** Logistic regression analysis for factors association with mental health outcomes.

	Anxiety OR (95% CI)	Depression OR (95% CI)	Psychological distress OR (95% CI)	Suicide attempts OR (95% CI)
**Age**	1.00 (0.97, 1.03)	0.99 (0.95, 1.05)	1.02 (0.98, 1.07)	0.99 (0.92, 1.08)
**Sex**				
Female	Ref.	Ref.	Ref.	Ref.
Male	**0.68 (0.55, 0.83)**	**0.68 (0.49, 0.96)**	**0.57 (0.40, 0.81)**	**0.44 (0.23, 0.86)**
**Marital status**				
Unmarried	Ref.	Ref.	Ref.	Ref.
Currently married	0.89 (0.69, 1.15)	0.95 (0.66, 1.36)	0.91 (0.67, 1.24)	0.90 (0.53, 1.52)
Divorced/Separated/Widowed	**3.34 (1.51, 7.37)**	**4.09 (1.91, 8.75)**	1.56 (0.65, 3.75)	4.95 (1.36, 18.05)
**Education**				
Illiterate/informal education	Ref.	Ref.	Ref.	Ref.
Grade 1–10	1.15 (0.77, 1.70)	0.91 (0.49, 1.67)	1.39 (0.99, 1.96)	1.42 (0.65, 3.09)
High school	1.28 (0.81, 2.03)	1.16 (0.60, 2.24)	**1.93 (1.27, 2.95)**	1.26 (0.64, 2.48)
Bachelors or higher	1.08 (0.61, 1.89)	1.14 (0.50, 2.57)	1.07 (0.59, 1.96)	0.86 (0.22, 3.39)
**Wealth quintile**				
Poorest	Ref.	Ref.	Ref.	Ref.
Poorer	**0.57 (0.37, 0.87)**	**0.46 (0.28, 0.77)**	**0.51 (0.30, 0.87)**	0.76 (0.39, 1.45)
Middle	**0.46 (0.29, 0.73)**	**0.38 (0.22, 0.65)**	**0.30 (0.16, 0.53)**	0.95 (0.54, 1.67)
Richer	**0.43 (0.26, 0.68)**	**0.33 (0.18, 0.62)**	**0.27 (0.14, 0.50)**	0.88 (0.48, 1.60)
Richest	**0.50 (0.30, 0.84)**	**0.33 (0.16, 0.67)**	**0.26 (0.14, 0.50)**	1.04 (0.49, 2.21)
**Place of residence**				
Rural	Ref.	Ref.	Ref.	Ref.
Urban	0.69 (0.47, 1.00)	0.86 (0.54, 1.39)	1.02 (0.67, 1.58)	1.12 (0.69, 1.84)
**Smoking**				
No	Ref.	Ref.	Ref.	Ref.
Yes	**2.37 (1.73, 3.25)**	**2.14 (1.47, 3.11)**	**1.84 (1.23, 2.77)**	1.90 (0.98, 3.69)
**Alcohol consumption in the last month**				
No	Ref.	Ref.	Ref.	Ref.
Yes	0.83 (0.61, 1.14)	1.25 (0.78, 1.98)	0.87 (0.58, 1.31)	1.12 (0.59, 2.11)
**Body Mass Index (BMI)**	1.00 (0.98, 1.03)	0.97 (0.92, 1.03)	0.98 (0.94, 1.02)	0.97 (0.90, 1.04)
**Adverse childhood experiences (ACEs)**				
No ACEs	Ref.	Ref.	Ref.	Ref.
One ACE	1.14 (0.91, 1.43)	1.02 (0.71, 1.47)	**1.57 (1.21, 2.03)**	1.90 (0.88, 4.09)
Two ACEs	1.22 (0.96, 1.56)	0.98 (0.65, 1.46)	**2.38 (1.75, 3.25)**	2.36 (0.99, 5.60)
Three ACEs	1.32 (0.96, 1.81)	1.19 (0.79, 1.79)	**2.28 (1.59, 3.27)**	2.85 (1.18, 6.90)
4+ ACEs	**2.72 (2.05, 3.62)**	**2.61 (1.74, 3.91)**	**4.67 (3.22, 6.77)**	**9.60 (4.44, 20.75)**
**Perceived social support score**	**0.97 (0.95, 0.99)**	0.95 (0.90, 1.00)	**0.92 (0.89, 0.95)**	**0.87 (0.82, 0.91)**
**Brief Resilience Scale score**	**0.79 (0.7, 0.91)**	**0.78 (0.65, 0.94)**	**0.79 (0.66, 0.94)**	**0.69 (0.54, 0.88)**
**Linear trend test (ACE score as continuous variable)**^a^				
OR per ACE unit	**1.23 (1.14, 1.32)**	**1.24 (1.11, 1.38)**	**1.43 (1.31, 1.56)**	**1.74 (1.44, 2.09)**
**Non-linearity test (quadratic term)**^b^				
Quadratic OR	**1.07 (1.02, 1.12)**	**1.12 (1.05, 1.20)**	1.00 (0.94, 1.06)	1.10 (0.98, 1.24)

OR, Odds ratio; CI, confidence interval.

All models adjusted for age, sex, marital status, education, wealth quintile, place of residence, current smoking, alcohol use, BMI, social support score, and resilience score. Survey weights applied using probability-weighted logistic regression.

Bold indicates statistically significant associations (p < 0.05) or 95% confidence intervals excluding the null value.

Note: Categorical estimates are from the primary multivariable model with ACE exposure as an ordinal categorical variable. Linear trend estimates are from models treating the cumulative ACE score as a continuous variable. Non-linearity was assessed by adding a quadratic term (ACE^2^) to the continuous model. A significant quadratic OR indicates an accelerating (rather than constant) rate of increase.

a Linear trend testing confirmed significant graded associations across all outcomes.

b Non-linearity testing indicated an accelerating association for anxiety and depression; associations for psychological distress and suicidal ideation were consistent with a linear pattern.

Male participants were less likely to experience depression compared with female participants (OR = 0.68; 95% CI: 0.49–0.96). Those divorced, separated, or widowed were more likely to experience depression (OR = 4.09; 95% CI: 1.91–8.75) compared with unmarried participants. Participants in higher wealth quintiles were less likely to experience depression than those in the poorest group. Current smokers were more likely to experience depression (OR = 2.14; 95% CI: 1.47–3.11) compared with non-smokers. Those with four or more ACEs were strongly associated with higher odds of depression (OR = 2.61; 95% CI: 1.74–3.91) compared to those with no ACEs. Those with higher resilience were associated with lower odds of experiencing depression (OR = 0.78; 95% CI: 0.65–0.94) (Table 4).

Male participants were less likely to experience psychological distress compared with female participants (OR = 0.57; 95% CI: 0.40–0.81). Participants with higher school education were more likely to experience psychological distress than those with no formal education (OR = 1.93; 95% CI: 1.27–2.95). Higher household wealth status was associated with lower odds of distress compared with the poorest group. Current smoking was associated with increased odds of distress (OR = 1.84; 95% CI: 1.23–2.77). Those with one or more ACEs, particularly four or more, were associated with markedly higher odds of psychological distress (OR = 4.67; 95% CI: 3.22–6.77) than those without ACEs. Higher perceived social support (OR = 0.92; 95% CI: 0.89–0.95) and resilience (OR = 0.79; 95% CI: 0.66–0.94) were associated with lower odds of experiencing distress (Table 4).

Male participants had lower odds of reporting suicide attempts compared with female participants (OR = 0.44; 95% CI: 0.23–0.86). Divorced, separated, or widowed participants were more likely to report suicide attempts than unmarried participants (OR = 4.95; 95% CI: 1.36–18.05). Those with higher exposure to ACEs were more likely to report suicide attempts, with those reporting four or more ACEs having the highest odds (OR = 9.60; 95% CI: 4.44–20.75). Higher perceived social support (OR = 0.87; 95% CI: 0.82–0.91) and resilience (OR = 0.69; 95% CI: 0.54–0.88) were associated with lower odds of reporting suicide attempts (Table 4).

Among the ACE categories, peer victimisation showed the strongest association with all four outcomes (anxiety OR = 2.36; depression OR = 2.94; psychological distress OR = 1.97; suicidal ideation OR = 2.42). Abuse was also associated with all outcomes (Table 5).

**Table 5:** Adverse childhood experience (ACE) domain-specific associations with mental health outcomes.

ACE domain	Anxiety OR (95% CI)	Depression OR (95% CI)	Psych. distress OR (95% CI)	Suicidal ideation OR (95% CI)
Abuse	**1.60 (1.28–1.99)**	**1.39 (1.09, 1.78)**	**1.92 (1.43, 2.59)**	**1.92 (1.25, 2.95)**
Neglect	**1.38 (1.06, 1.79)**	**1.25 (0.85, 1.83)**	1.30 (0.95, 1.77)	1.46 (0.92, 2.31)
Household dysfunction	0.95 (0.76, 1.18)	0.92 (0.66, 1.29)	**1.69 (1.27, 2.27)**	1.67 (0.99, 2.81)
Peer victimisation	**2.36 (1.74, 3.20)**	**2.94 (2.06, 4.18)**	**1.97 (1.45, 2.68)**	**2.42 (1.36, 4.32)**

ACE domains: Abuse = verbal, physical, and sexual abuse; Neglect = physical and emotional neglect; Household dysfunction = parental divorce, parental death, parental mental illness, household substance abuse, household dysfunction, and parental incarceration; Peer victimisation = bullying.

Adjusted for age, sex, marital status, education, wealth quintile, place of residence, current smoking, alcohol use, BMI, social support score, and resilience score.

Bold indicates statistically significant associations (p < 0.05) or 95% confidence intervals excluding the null value.

The decomposition of association between ACEs and mental health outcomes revealed that perceived social support accounted for 14.2% of the association between ACEs and anxiety (OR = 1.05; 95% CI: 1.03, 1.08), 26.1% of the association between ACEs and depression (OR = 1.09; 95% CI: 1.04, 1.15), 13.8% of the association between ACEs and psychological distress (OR = 1.14; 95% CI: 1.09, 1.19), and 21.6% of the association between ACEs and suicide attempts (OR = 1.40; 95% CI: 1.24, 1.59). Lower resilience accounted for 3.1% of the association between ACEs and suicide attempts (OR = 1.05; 95% CI: 1.01, 1.10) (Table 6).

**Table 6:** Estimates of direct and indirect effects mediated through resilience and social support on anxiety, depression, psychological distress and suicide attempts.

	OR (95% CI)			% of total association^a^	
	TA	DA	IA	%DA	%IA
**Anxiety**					
*Mediation by resilience*					
No ACEs	1	1	1		
Presence of ACEs	1.44 (1.26, 1.65)	1.43 (1.25, 1.63)	1.01 (1.00, 1.02)	97.9	2.1
*Mediation by social support*					
No ACEs	1	1	1		
Presence of ACEs	1.44 (1.26, 1.65)	1.37 (1.20, 1.56)	**1.05 (1.03, 1.08)**	85.8	14.2
**Depression**					
*Mediation by resilience*					
No ACEs	1	1	1		
Presence of ACEs	1.41 (1.10, 1.80)	1.39 (1.09, 1.78)	1.02 (1.00, 1.03)	95.6	4.4
*Mediation by social support*					
No ACEs	1	1	1		
Presence of ACEs	1.41 (1.10, 1.81)	1.29 (1.01, 1.65)	**1.09 (1.04, 1.15)**	73.9	26.1
**Psychological distress**					
*Mediation by resilience*					
No ACEs	1	1	1		
Presence of ACEs	2.62 (2.09, 3.28)	2.58 (2.06, 3.23)	1.02 (1.00, 1.03)	98.4	1.6
*Mediation by social support*					
No ACEs	1	1	1		
Presence of ACEs	2.61 (2.09, 3.26)	2.28 (1.83, 2.86)	**1.14 (1.09, 1.19)**	86.2	13.8
**Suicidal attempts**					
*Mediation by resilience*					
No ACEs	1	1	1		
Presence of ACEs	4.90 (2.63, 9.14)	4.67 (2.50, 8.70)	**1.05 (1.01, 1.10)**	96.9	3.1
*Mediation by social support*					
No ACEs	1	1	1		
Presence of ACEs	4.83 (2.64, 8.84)	3.44 (1.86, 6.36)	**1.40 (1.24, 1.59)**	78.4	21.6

TA, Total Association; DA, Direct Association; IA, Indirect Association.

Bold indicates statistically significant associations (p < 0.05) or 95% confidence intervals excluding the null value.

a % of total association refers to the changes in beta-coefficients in the models i.e., natural logarithm of odds ratios (ORs). The results are presented in terms of ORs and 95% confidence intervals (95% CI) for ease of interpretation.

## Discussion

This population-based household survey of young people in Nepal provides an estimate of population burden of ACEs and their associations with mental health conditions. Over 62% of young people aged 16–29 years reported exposure to at least one ACE, with more than 10% experiencing four or more. Nearly 4 in 10 young people experienced at least one mental health problem, and the risk escalated with increasing ACE exposure. Female sex, socioeconomic disadvantage, relationship disruptions, and current smoking were associated with increased mental health risks. ACEs exposure shows a clear graded associations with anxiety, depression, psychological distress, and suicide attempts. Peer victimisation and abuse had the strongest association with all four outcomes (Table 5). Psychosocial protective factors include resilience and social support.

Nepal's ACE prevalence falls within the range observed across other LMICs, though regional variation is substantial.^34^, ^35^, ^36^ Among young adults, India reports notably high rates, with 71.5% of young women (18–25 years) experiencing at least one ACE and 16% reporting severe exposure (four or more).^34^ Kenya shows similarly elevated prevalence at 65.8% for any ACE exposure and 19.3% for severe ACEs.^35^ By contrast, Indonesia and Vietnam demonstrate considerably lower rates, with only 40.2% and 36.9% of adolescents experiencing any ACE, and just 7.6% and 5.2% reporting severe exposure, respectively.^35^ A global meta-analysis of 206 studies across 22 countries found intermediate rates—approximately 40% experiencing at least one ACE and 16.1% reporting four or more.^36^ These findings suggest that ACE burden in Nepal reflects broader patterns in South and Southeast Asia, where country-level differences in socioeconomic conditions, health systems, and social structures likely drive significant variation in both prevalence and severity.

Studies collectively show a high prevalence of childhood adversity and its strong link with mental health outcomes, underscoring the widespread nature of childhood adversity in LMICs.^37^^,^^38^ Our findings, based on a large population-based sample, underscore the cumulative psychological harm associated with childhood adversity among young people in Nepal. The consistency of associations observed across anxiety, depression, and psychological distress suggests that ACE exposure may influence multiple dimensions of emotional distress rather than a single disorder-specific pathway. Notably, peer victimisation demonstrated the strongest associations across all mental health outcomes, highlighting bullying as a potentially under-recognised but important contributor to youth mental health problems in Nepal. The findings suggest that the overall ACE framework retains relevance in the Nepali context, while also highlighting locally salient adversities such as household substance use, household violence, and physical neglect.

Our findings demonstrated heightened vulnerability among female participants compared to male participants, consistent with previous studies from Nepal.^14^^,^^18^, ^19^, ^20^ Among female participants in particular, these vulnerabilities are more intensified, with heightened risk of mental perhaps linked to gender discrimination, gender-based violence and restrictive socio-cultural norms.^39^, ^40^, ^41^ Additionally, our findings also corroborate evidence on the relationship between poverty, relationship breakdown, low social support, and mental health risks.^18^^,^^42^

Our findings also indicated that young people who smoke and have high ACE scores are more likely to experience anxiety, depression, and psychological distress, suggesting a bidirectional relationship between risky behaviours and mental health. In Nepal, tobacco and substance use among adolescents and youth is common, with 11–25% currently smoking and initiation often occurring in early adolescence potentially serving to cope with stress or adverse familial and social environments.^43^ Given these patterns, incorporating age-appropriate content on ACEs, emotional regulation, and healthy behaviours, including tobacco cessation and healthy lifestyles, into the school curriculum could provide a critical, scalable platform for early intervention among Nepali adolescents.

Suicide attempts, although less common than other mental health outcomes, were strongly associated with multiple ACEs, with individuals exposed to four or more ACEs being at highest risk. Female participants, those experiencing relationship disruptions, and individuals with lower social support and resilience were particularly vulnerable. In Nepal, current police data report over 7000 suicide deaths annually among young people,^44^ and the National Mental Health Survey 2020 also showed suicidality to be highest among those aged 18–29 years.^11^ Despite these patterns, Nepal still lacks a dedicated national suicide prevention strategy.^45^

To address the rising rates of suicide among youth in Nepal, systematic early identification of high-risk children and referral could be critical, alongside the implementation of comprehensive suicide prevention strategies, including school- and community-based awareness, crisis intervention, and accessible psychosocial support networks. Additionally, the modifiable protective psychosocial factors identified in this study suggest that school-based programs, including peer support initiatives and family-based interventions (e.g., parenting interventions), could provide scalable and low-cost strategies to mitigate the high rates of suicide and the increasing burden of mental health conditions among young people.

In Nepal, where mental health services remain limited and are largely concentrated in urban centres, leveraging existing community-based platforms such as Female Community Health Volunteers (FCHVs), primary healthcare systems, and schools might offer a feasible pathway for implementing ACE-informed and resilience-focused interventions. Building on recent efforts by Nepal's Ministry of Health to integrate mental health into primary care, including developing evidence-based training packages^46^ and inclusion of common mental health conditions within the basic health service package,^47^ there is an opportunity to expand these initiatives to better address the needs of adolescents and young adults. These could include capacity building programs that train community members to recognise trauma-related mental health problems^48^ or implement school-based group psychosocial (resilience-building) interventions^49^^,^^50^ and implement public awareness campaigns regarding mental health and ACEs prevention.^51^

Given that young people from Nepal and other South Asian countries are a growing part of the global workforce, targeted programs to strengthen mental health literacy, healthcare access, and community support in similar settings are increasingly globally important.^52^

This study provides population-based evidence on ACE exposure and mental health outcomes among young people in Nepal using a multi-stage probability sampling design. The large sample size and inclusion of diverse geographic and socioeconomic contexts strengthen the robustness of the estimates. This study simultaneously examines cumulative ACE exposure and psychosocial protective factors, offering a more comprehensive understanding of risk and resilience within a South Asian context. Standardised and widely used instruments were employed for key mental health outcomes, enhancing comparability with existing literature. Formal interaction testing, non-linearity assessment, ACE domain-specific analyses, and sensitivity analyses using alternative outcome thresholds further strengthen the analytical rigour of the findings.

However, several limitations should be considered. The cross-sectional design precludes causal inference, and the combination of retrospective ACE reporting with contemporaneous mental health assessment limits temporal interpretation. Self-reported measures of ACEs and sensitive outcomes such as suicide attempts may be subject to recall bias, social desirability bias, and potential underreporting despite efforts to ensure privacy during interviews. While PHQ-9, GAD-7, and K-6 have been validated in Nepal or comparable South Asian setting, other measures including BRS, PSSS, and ACE items have not been formally validated among Nepali youth, which may introduce some measurement uncertainty.

Although provinces were selected using probability sampling, only three of Nepal's seven provinces were included. While sampling weights were applied, regional heterogeneity across all provinces may not be fully captured, and findings should be interpreted as population-based estimates under the sampling design rather than strictly nationally exhaustive. The household-based sampling approach may have excluded highly vulnerable populations such as homeless, migrant, or institutionalised youth. Survivorship bias may influence estimates of suicide attempts, as individuals who died by suicide were not captured, and observed sex differences may partly reflect differential survival.

The study population spans late adolescence and young adulthood, and although age was adjusted for in the analysis, developmental differences across this range may influence exposure and outcomes. The mediation analyses explored the extent to which resilience and perceived social support explain the association between ACEs and mental health outcomes. Given the cross-sectional design, these analyses are exploratory and intended to decompose potential pathways, rather than to infer causal effects.

Future studies using longitudinal designs are recommended to examine the long-term mental health outcomes associated with the expanded ACEs framework, including examination of potential mediation pathways and age-stratified analyses to explore developmental differences in ACE exposure and mental health outcomes. Mixed-methods research would also help elucidate how contextual factors, such as cultural, social, and gender norms, shape the ACEs-mental health relationship, areas that were beyond the scope of this analysis.

In conclusion, our study highlights that over 60% of young people aged 16–29 years reported exposure to at least one ACE, with more than 10% experiencing four or more in Nepal. Nearly four in ten individuals experienced at least one mental health condition—anxiety, depression, psychological distress, or suicide attempts—with risk escalating in a cumulative manner with increasing numbers of ACEs, particularly household substance use, household violence, and physical neglect. The protective roles of social support and resilience highlight potential intervention targets. These findings underscore critical gaps in ACE-related prevention, diagnosis, and treatment for young people in Nepal and provide an evidence base for regional strategies in other LMICs confronting similar challenges of poverty, social exclusion, and limited mental health infrastructure.

## Contributors

ST conceptualised the study, developed the study instruments, supervised fieldwork, verified the data, and interpreted the findings. SG led the statistical analysis. ST and SG had full access to all study data and took responsibility for the integrity of the data and the accuracy of the data analysis. ST drafted the first version of the manuscript. NR, SG, KYA, SB, DGF, AB, and JA critically revised the manuscript for important intellectual content. SB and DGF contributed to study design, field implementation, and interpretation of findings, with particular attention to cultural relevance, ethical considerations, and contextual appropriateness. All authors reviewed and approved the final version of the manuscript and take responsibility for its content.

## Data sharing statement

The data that support the findings of this study are not publicly available due to the sensitive nature of the information collected and the need to protect participant confidentiality. De-identified data may be made available upon reasonable request to the corresponding author, subject to approval by the relevant ethics committees and data-sharing agreements.

## Editor note

The Lancet Group takes a neutral position with respect to territorial claims in published maps and institutional affiliations.

## Declaration of interests

The authors declare no conflict of interests.
