# Supplementary material for: Adverse childhood experiences, resilience and mental health among young people aged 16–29 years in Nepal: a population-based household survey (2025)
Source: Lancet Reg Health Southeast Asia. 2026 Jun 15;51:100803. doi: 10.1016/j.lansea.2026.100803 (PMC13285757; doi:10.1016/j.lansea.2026.100803)
Supplement: Supplementary Tables [file mmc1.docx]

**Supplementary tables**

Effect modification by sex and wealth quintile was assessed by introducing multiplicative interaction terms (ACE × sex; ACE × wealth quintile) and testing their joint significance using adjusted Wald tests. Age-group heterogeneity was evaluated by stratifying participants into adolescents (16–19 years) and young adults (20–29 years), with a formal interaction test (ACE × age group) conducted prior to stratified analyses. A sensitivity analysis was conducted for the anxiety outcome using the standard clinical GAD-7 cutoff of ≥10 to assess robustness of findings obtained at the primary threshold of ≥6.

Formal interaction testing revealed no statistically significant effect modification by sex (all outcomes p>0.47) or wealth quintile (all outcomes p>0.18), indicating that the graded association between ACE exposure and mental health outcomes was consistent across these subgroups (Table S1). Age-group interaction tests also did not reach statistical significance for any outcome (all p>0.10). Stratified analyses confirmed consistent graded associations in both adolescents (16–19 years) and young adults (20–29 years), with 4+ ACEs associated with substantially elevated odds across all outcomes in both developmental groups (Table S2). Among adolescents, a notably strong association was observed between 4+ ACEs and suicidal ideation (OR=28.6, 95% CI 7.5–108.8); however, the wide confidence interval reflects small cell counts in this subgroup, and this estimate should be interpreted with caution.

A sensitivity analysis using the standard clinical GAD-7 threshold of ≥10 yielded consistent graded associations between cumulative ACE exposure and anxiety, with statistically significant associations from two or more ACEs (Table S3), indicating that primary findings were not an artefact of the lower detection threshold employed.

**Table S1. Effect modification testing: ACE × sex and ACE × wealth quintile**

Adjusted Wald tests for multiplicative interaction terms (n=6,039)

| **Interaction** | **Anxiety** | **Depression** | **Psychological distress** | **Suicidal ideation** |
| --- | --- | --- | --- | --- |
| **ACE exposure × sex** | | | | |
| F-statistic (df) | F (4, 36) = 0.74 | F (4, 36) = 0.90 | F (4, 36) = 0.67 | F (4, 36) = 0.58 |
| p-value | 0.573 | 0.475 | 0.614 | 0.679 |
| **ACE exposure × wealth quintile** | | | | |
| F-statistic (df) | F (16, 24) = 0.65 | F (16, 24) = 1.31 | F (16,2 4) =1.28 | F (16, 24) = 0.97 |
| p-value | 0.809 | 0.266 | 0.183 | 0.470 |

**Table S2. Age-stratified associations between cumulative ACEs and mental health outcomes**

Survey-weighted logistic regression. OR (95% CI). Reference: No ACEs. Adolescents: 16, 19 years (n=2,265); Young adults: 20, 29 years (n=3,462).

| **ACE exposure** | **Anxiety** | | **Depression** | | **Psychological distress** | | **Suicidal ideation** | |
| --- | --- | --- | --- | --- | --- | --- | --- | --- |
|  | **16-19 yrs** | **20-29 yrs** | **16-19 yrs** | **20-29 yrs** | **16-19 yrs** | **20-29 yrs** | **16-19 yrs** | **20-29 yrs** |
| One ACE | **1.48 (1.11, 1.97)** | 0.99 (0.76, 1.30) | 1.03 (0.65, 1.64) | 1.06 (0.63, 1.77) | 1.46 (0.92, 2.32) | **1.81 (1.25, 2.62)** | 2.49 (0.48, 12.8) | 1.67 (0.72, 3.85) |
| Two ACEs | 1.31 (0.91, 1.88) | 1.09 (0.81, 1.47) | 1.03 (0.59, 1.80) | 0.93 (0.59, 1.47) | 1.56 (0.94, 2.59) | **3.10 (1.98, 4.85)** | 2.85 (0.52, 15.5) | 1.91 (0.69, 5.24) |
| Three ACEs | 1.25 (0.71, 2.19) | 1.40 (1.03, 1.90) | 0.86 (0.38, 1.97) | 1.34 (0.81, 2.20) | 1.96 (0.96, 4.00) | **2.88 (1.75, 4.74)** | **7.85 (2.03, 30.4)** | 1.39 (0.50, 3.85) |
| 4+ ACEs | **3.31 (2.12, 5.17)** | **2.35 (1.75, 3.18)** | **2.64 (1.44, 4.83)** | 2.43 (1.56, 3.79) | **4.94 (2.75, 8.85)** | **5.02 (3.16, 7.98)** | **28.6 (7.52, 109)** | **4.62 (2.11, 10.1)** |
| **Age × ACE interaction test (adjusted Wald test)** | | | | | | | | |
| F (df); p | F (5, 35) = 2.01; p=0.102 | | F (5, 35) = 0.38; p=0.856 | | F (5, 35) = 1.34; p=0.270 | | F (5, 35) = 1.50; p=0.216 | |

*All models adjusted for age, sex (where applicable), marital status, education, wealth quintile, place of residence, current smoking, alcohol use, BMI, social support score, and resilience score. Among adolescents, the divorced/separated/widowed marital category was omitted from models due to perfect prediction in the small subgroup.*

**Table S3. Sensitivity analysis: anxiety at stricter GAD-7 cutoff (≥10)**

Survey-weighted logistic regression. OR (95% CI). Reference: No ACEs. n=6,039.

| **ACE exposure** | **OR (95% CI)** | **p-value** |
| --- | --- | --- |
| One ACE | 1.07 (0.75, 1.54) | 0.702 |
| Two ACEs | **1.74 (1.08, 2.80)** | 0.023 |
| Three ACEs | **1.70 (1.06, 2.72)** | 0.029 |
| 4+ ACEs | **2.63 (1.65, 4.20)** | <0.001 |

**Table S4. Tetrachoric correlations and weighted comorbidity between mental health outcomes**

Tetrachoric correlations computed for binary outcomes. Comorbidity proportions are weighted. n=6,039.

|  | **Anxiety** | **Depression** | **Psychological distress** | **Suicidal ideation** |
| --- | --- | --- | --- | --- |
| Anxiety | - |  |  |  |
| Depression | 0.82*** | - |  |  |
| Psych. distress | 0.77*** | 0.73*** | - |  |
| Suicidal ideation | 0.47*** | 0.49*** | 0.61*** | - |
| **Weighted comorbidity proportions (% of total sample)** | | | | |
| Anxiety + depression | 5.6% (95% CI: 5.0, 6.2) | | | |
| Anxiety + psychological distress | 8.1% (95% CI: 7.5, 8.8) | | | |
| Depression + psychological distress | 3.4% (95% CI: 3.0, 3.9) | | | |
| All three outcomes | 3.3% (95% CI: 2.9, 3.8) | | | |

**** p<0.001 for all tetrachoric correlations. High correlations between anxiety, depression, and psychological distress (r=0.73, 0.82) indicate substantial shared latent variance. These findings are discussed in the Limitations section. Suicidal ideation showed comparatively lower correlations with the three affective outcomes (r=0.47, 0.61), suggesting greater distinctiveness as an outcome construct.*
